# Negative predictive value of fecal immunochemical testing in significant bowel disease screening: a systematic review and meta-analysis

**DOI:** 10.1097/JS9.0000000000001844

**Published:** 2024-06-26

**Authors:** Zhen Junhai, Liao Fei, Zhang Jixiang, Xie Huabing, Tan Cheng, Dong Weiguo

**Affiliations:** a Department of General Practice, Renmin Hospital of Wuhan University; b Department of Gastroenterology, Renmin Hospital of Wuhan University, Wuhan, Hubei Province, People’s Republic of China

**Keywords:** fecal immunochemical tests, primary care, significant bowel disease, screening

## Abstract

**Objectives:**

General practitioners (GPs) must assess significant bowel disease (SBD) in patients with lower bowel symptoms during primary care. Studies have evaluated the efficacy of fecal immunochemical testing (FIT) for SBD screening. However, the effectiveness of FIT remains controversial. This study aimed to investigate the value of FIT in SBD screening.

**Methods:**

PubMed, the Cochrane Database, and EMBASE were systematically searched. Studies that estimated FIT values in screening for SBD among patients with lower bowel symptoms were included. Sensitivity, specificity, negative likelihood ratio (NLR), positive likelihood ratio (PLR), diagnostic odds ratio (DOR), and negative predictive value (NPV) were calculated. Additionally, the pooled area under the summary receiver operating characteristic (SROC) curve was analyzed.

**Results:**

A total of 8615 patients with lower bowel symptoms who underwent FIT and colonoscopy to screen for SBD were enrolled and assessed in this meta-analysis; of these, 1226 patients were ultimately diagnosed with SBD. The pooled sensitivity, specificity, PLR, NLR, DOR, and NPV of FIT in SBD screening were 0.65 (95% CI: 0.50–0.78), 0.85 (95% CI: 0.72–0.92), 4.2 (95% CI: 2.60–6.90), 0.41 (95% CI: 0.29–0.58), 10 (95% CI: 6–17), and 0.90 (95% CI: 0.87–0.94), respectively. Besides, the pooled SROC was 0.82 (95% CI: 0.78–0.85).

**Conclusions:**

This study indicates that the FIT provides a favorable NPV for SBD screening and could be a valuable technique for GPs to rule out SBD in primary care. At the same time, GPs need to remain vigilant and refer patients to gastroenterologists when necessary.

## Introduction

Highlights

- It is essential for general practitioners to detect significant bowel disease (SBD) among patients with lower bowel symptoms in order to receive therapy like timely surgery.
- Studies have evaluated the efficacy of fecal immunochemical testing (FIT) in identifying SBD, however, the performance of FIT still remains controversial.
- This meta-analysis incorporating nine studies with up to 8615 patients suffered from lower bowel symptoms, this study found the negative predictive value of FIT in SBD screening was high to 0.90 (95% CI: 0.87–0.94).
- FIT provides a favorable negative predictive value in SBD screening, it could be a valuable technique for general practitioners to rule out SBD in primary care.

Patients with complaints of lower bowel symptoms are common in primary care settings^1^; however, only 22–37% of patients with lower bowel symptoms are eventually diagnosed with significant bowel diseases (SBD), including colorectal cancer (CRC), high-risk adenoma (HRA), and inflammatory bowel disease (IBD)^2^. Moreover, the lower bowel symptoms reported for gut diseases overlap considerably^3^. Previous studies concluded that diagnostic strategies based on symptoms or signs alone are insufficient for detecting underlying pathologies in general practice^4,5^. A recent retrospective cohort study showed that delays in referral from general practitioners (GPs) worsen the survival of patients with CRC if erroneously classified as benign conditions^6^. Therefore, assessing SBD in patients with lower bowel symptoms is a critical challenge for GPs.

Diagnostic tests assist GPs in screening for SBD, one of which is the widely used fecal immunochemical testing (FIT). FIT measures intact fecal hemoglobin concentration, and the 2015 National Institute of Health and Care Excellence (NICE) recommends quantitative FIT for CRC screening in primary care^7^. However, in symptomatic patients, besides CRC, GPs should also be vigilant for other SBDs, such as HRA and IBD. Patients with HRA and IBD require a referral for further investigation. Most published studies have focused on CRC screening alone and not on the efficacy of FIT in screening for SBD. In recent years, some studies have demonstrated that FIT has a good capacity for SBD screening with a high negative predictive value (NPV)^8–10^. Conversely, a recently published study found that the FIT-only referral pathway missed >23% of SBD^11^. Thus, the performance and effect of FIT in SBD screening remain debatable, and guidelines for SBD screening in primary care settings are still lacking. Therefore, this meta-analysis aimed to systematically investigate the value of FIT in screening for SBD.

## Methods

### Protocol and guidance

This work was reported in accordance with the preferred reporting items for systematic reviews and meta-analyses (PRISMA, Supplemental Digital Content 1, http://links.lww.com/JS9/C890, Supplemental Digital Content 2, http://links.lww.com/JS9/C891)^12^ and assessing the methodological quality of systematic reviews (AMSTAR, Supplemental Digital Content 3, http://links.lww.com/JS9/C892) guidelines^13^. The study protocol was registered in PROSPERO (https://www.york.ac.uk/inst/crd)^14^, an international database of prospectively registered systematic reviews.

### Literature search strategy

Two authors systematically searched the electronic databases MEDLINE, Embase, and Cochrane independently, according to the Preferred Reporting Items for Systematic Reviews and Meta-Analyses statement, for articles published from the inception of the databases to 31 December 2023. The literature search was restricted to English. We used the following keywords: ‘significant bowel disease’, ‘significant colorectal disease’, ‘fecal immunochemical test’, ‘immunochemical fecal occult blood test’, ‘fecal immunochemical test’, ‘FIT’, ‘occult blood’, ‘fecal occult blood test’, ‘fob’, ‘fobt’, and ‘ifobt’, etc. Any disagreements between the two authors during the database searches were resolved through discussion, and a third author participated in decision-making when the discussion failed to resolve the disagreements. One author performed the search, followed by the other authors. EndNote software (version X9.0) was used to exclude duplicate studies.

### Eligibility criteria

Literature was selected for the systematic review based on the following eligibility criteria^1^: studies designed to evaluate the ability of FIT in SBD screening among adult patients with lower bowel symptoms^2^; true positive (TP), true negative (TN), false negative (FN), and false positive (FP) results were all complete or could be inferred from other data; and^3^ all symptomatic patients underwent endoscopy to determine whether they were diagnosed with SBD. The exclusion criteria were as follows: systematic reviews or meta-analyses, case reports, studies with experimental designs, studies involving pediatric patients, editorials or letters, and repeated reports.

### Data extraction

Two authors independently read and assessed the articles according to the eligibility and exclusion criteria of our meta-analysis, and the decisions were reviewed by a third author. Data from the included studies were extracted using a standardized data extraction form. As previously stated, a third author joined the consensus meetings for any unresolved discrepancies or disagreements. The selected studies were carefully and thoroughly reviewed. The extracted data included the following: 1) basic study characteristics: the first author’s name of the study, publication year, country, setting, type of study design, age interval of the patients, total SBD cases in each study, and definition of SBD; and 2) other vital parameters: TP, FP, TN, FN, sensitivity, specificity, NPV, and FIT cutoff value in each study. For any enrolled study with missing data, the corresponding author was contacted via e-mail to obtain additional information.

### Methodological quality assessment

The methodological quality of the included studies was assessed according to the Quality Assessment of Diagnostic Accuracy Studies 2 (QUADAS-2) guidelines^15^. Two authors independently assessed the methodological quality of each study and a third author was invited to resolve any disagreements. The QUADAS-2 scale consists of risk of bias and applicability concerns. For risk of bias, studies were judged based on patient selection, index test, reference standard, and flow and timing. For applicability concerns, the studies were judged based on patient selection, index tests, and reference standards. The seven items were categorized as high, low, or unclear risk.

### Statistical analysis

STATA statistical software V1.4 (Stata Corporation) was used to produce forest plots and measure the sensitivity, specificity, negative likelihood ratio (NLR), positive likelihood ratio (PLR), diagnostic odds ratio (DOR), and NPV. The Fagan nomogram and pooled area under the summary receiver operating characteristic (SROC) curve were used to evaluate the performance of FIT in SBD screening. Heterogeneity among studies was measured using inconsistency factor (*I* ^2^) statistics and the Cochran-*Q* test. A random-effects model was used to calculate the pooled effect size if statistical heterogeneity existed (*P*<0.05, *I* ^2^ >50); otherwise, a fixed-effects model was used. We further conducted subgroup analyses to identify the sources of heterogeneity. Deeks’ funnel plot was used to assess publication bias.

## Results

### Literature search results

**Figure 1:**
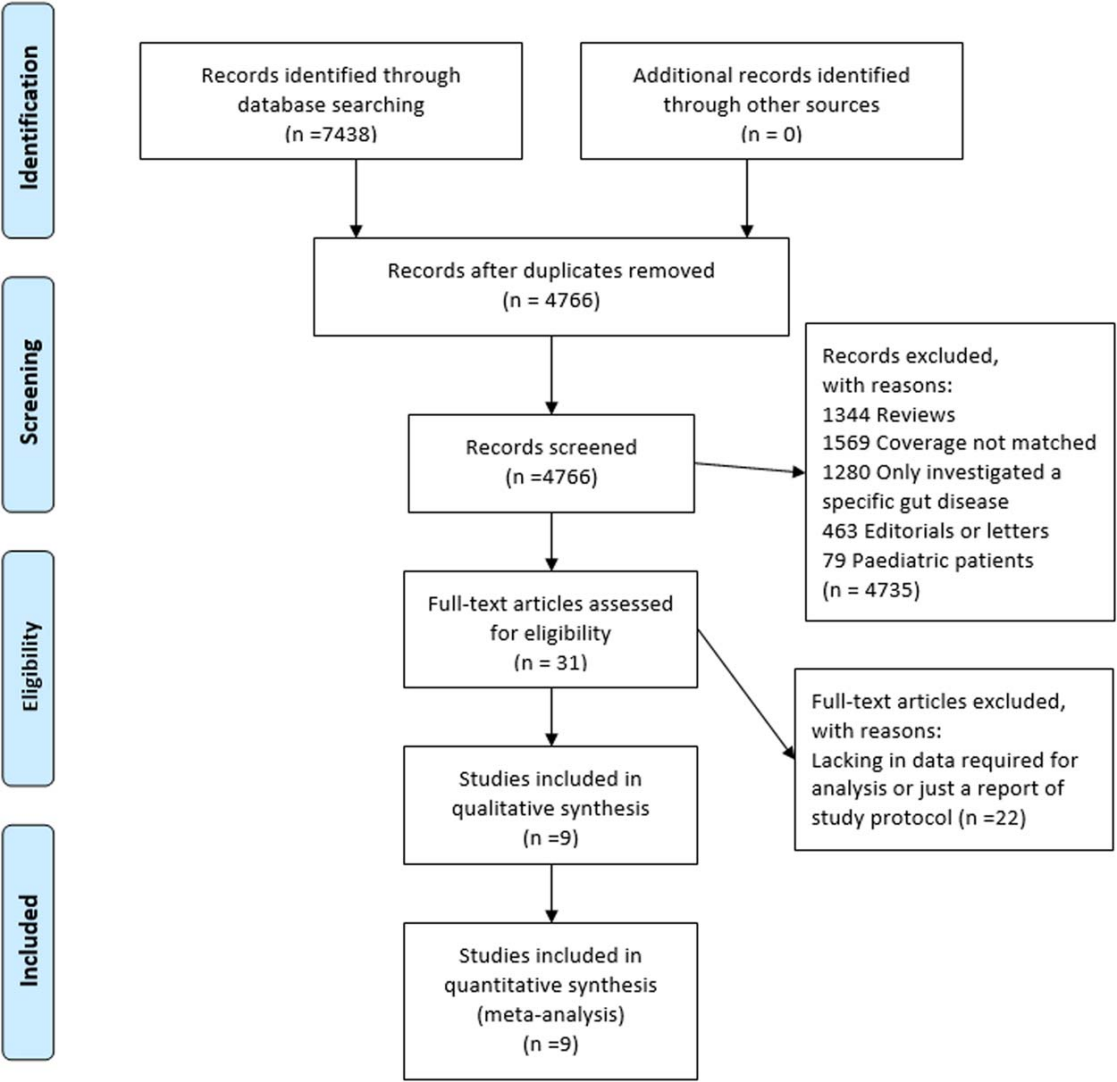
PRISMA flow diagram depicting the study selection process.

We identified 4766 references after removing duplicate articles from the database search. After a careful review of titles and abstracts, 4735 studies were excluded because they were systematic reviews, editorials, letters, consisted of pediatric patients, investigated only a specific gut disease, or did not match coverage. Of the remaining studies, 31 were read in full, and 22 additional studies were excluded because of a lack of relevant data or report of the study protocol. Finally, nine studies were included in the meta-analysis. Figure 1 shows the detailed PRISMA flowchart.

### Characteristics of the enrolled trials

Detailed characteristics of the nine included studies are shown in Table 1 and Supplemental Table 1 (Supplemental Digital Content 4, http://links.lww.com/JS9/C893). The publication years of the studies ranged from 2013 to 2023; six were performed in the UK^8–11,16,17^, and the remaining three originated from the Netherlands^18^, Australia^19^ and China^20^. Five studies were conducted in primary care^8–10,17,18^, and the other four were conducted in secondary care^11,16,19,20^; however, the patients were referred by their GPs from primary care. All studies were prospective observational cohort studies, and the total number of enrolled studies ranged from 201 to 4137, whereas the number of SBD cases ranged from 20 to 328. Regarding the definition of SBD, most studies regarded CRC, HRA, and IBD as SBD^8–11,17,19^; one study also included lower-risk adenoma (LRA) in the definition^16^; Elias *et al*.^18^ included diverticulitis, CRC, HRA, and IBD in the definition; and the study conducted by Zhu *et al*.^20^ included advanced neoplasia, active IBD, or bowel inflammation induced by other reasons. Regarding the FIT cut-off values, Mowat *et al*.^9^ used cut-off values of 0 and 10 μg Hb/g to evaluate the capacity of FIT in SBD screening and found that 10 µg Hb/g had a higher value. Another study utilized different cut-off values, including 7, 10, 20, and 50 µg Hb/g, and 7 µg Hb/g, which outperformed the other values^10^. The study performed by Zhu *et al*.^20^ used 22.5 and 50 ng Hb/ml as cut-off values and demonstrated that the ideal FIT cut-off for SBD screening was 22.5 ng Hb/ml. The remaining studies used a single cutoff value for FIT, three studies reported 10 µg Hb/g^8,11,17^, and the other three reported 51 ng Hb/g (16), 0 (19), and 6 (18) μg Hb/g. Five studies used FIT alone to explore the ability of FIT in SBD screening^8–11,16^, whereas four studies further analyzed the data by combining FIT with other parameters, such as routine clinical data^18,19^, fecal calprotectin, or the FAST score (FIT + age + sex test score)^17^. The sensitivity, specificity, and NPV of FIT for screening SBD were 17–97%, 26–99%, and 67–97%, respectively.

**Table 1:** Characteristics of the included studies.

Study	Country	Setting	Study design	Age interval (years)	Total cases	SBD cases	Definition of SBD
P.J. McDonald 2013	UK	Secondary care	Prospective observational cohort study	16–80	280	86	CRC, LRA, HRA, IBD
Craig Mowat 2016	UK	Primary care	Prospective observational cohort study	16–90	755	102	CRC, HRA, IBD
Sjoerd G. Elias 2016	Netherlands	Primary care	Prospective observational cohort study	19–92	810	141	CRC, HRA, IBD, Diverticulitis
Brian D Nicholson 2019	UK	Primary care	Prospective observational cohort study	19–93	238	20	CRC, HRA, IBD
Scott MacDonald 2022	UK	Secondary care	Prospective observational cohort study	Mean age: 62	4137	328	CRC, HRA, IBD
Waite MMA 2022^8^	UK	Primary care	Prospective observational cohort study	18–82	319	32	CRC, HRA, IBD
Jayne Digby 2019	UK	Primary care	Prospective observational cohort study	NA	1447	296	CRC, HRA, IBD
Anton R. Lord 2018	Australia	Secondary care	Prospective observational cohort study	54.3±13.8	428	114	CRC, HRA, IBD
Min Zhu 2022	China	Secondary care	Prospective observational cohort study	Mean age: 56	201	107	Advanced neoplasia, Active IBD or bowel inflammation for other causes

CRC, colorectal cancer; HRA, higher-risk adenoma; IBD, inflammatory bowel disease; LRA, lower-risk adenoma; SBD, significant bowel disease.

### Results of methodological quality evaluation

For the methodological quality evaluation of the nine included studies, six were judged as having a low risk of bias in the item of patient selection^9–11,16,18,20^, two studies^17,19^ had an unclear risk, and one study^8^ had a high-risk. All the enrolled studies had a low risk of bias in the index test, reference standard, flow, and timing. All studies were judged to have low applicability concerns. Overall, most included studies were of high quality (see details in Supplemental Table 2, Supplemental Digital Content 4, http://links.lww.com/JS9/C893).

### SBD screening performance of FIT

**Figure 2:**
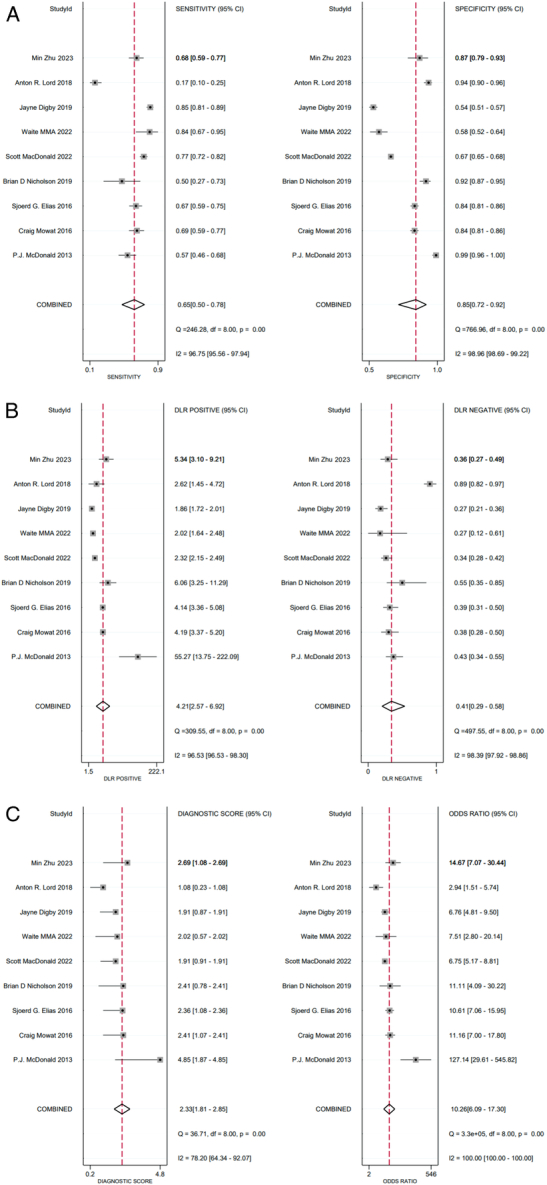
A, Forest plot of the sensitivity (SEN) and specificity (SPE) of FIT in SBD screening; the pooled SEN and SPE were 0.65 (95% CI: 0.50–0.78) and 0.85 (95% CI: 0.72–0.92), respectively. B, Forest plot of the positive likelihood ratio (PLR), negative likelihood ratio (NLR) of FIT in SBD screening; the pooled PLR and NLR were 4.2 (95% CI: 2.60–6.90) and 0.41 (95% CI: 0.29–0.58), respectively. C, Forest plot of the diagnostic score and diagnostic odds ratio (DOR) of FIT in SBD screening; the pooled diagnostic score and DOR were 2.33 (95% CI: 1.81–2.85) and 10 (95% CI: 6–17), respectively.

**Figure 3:**
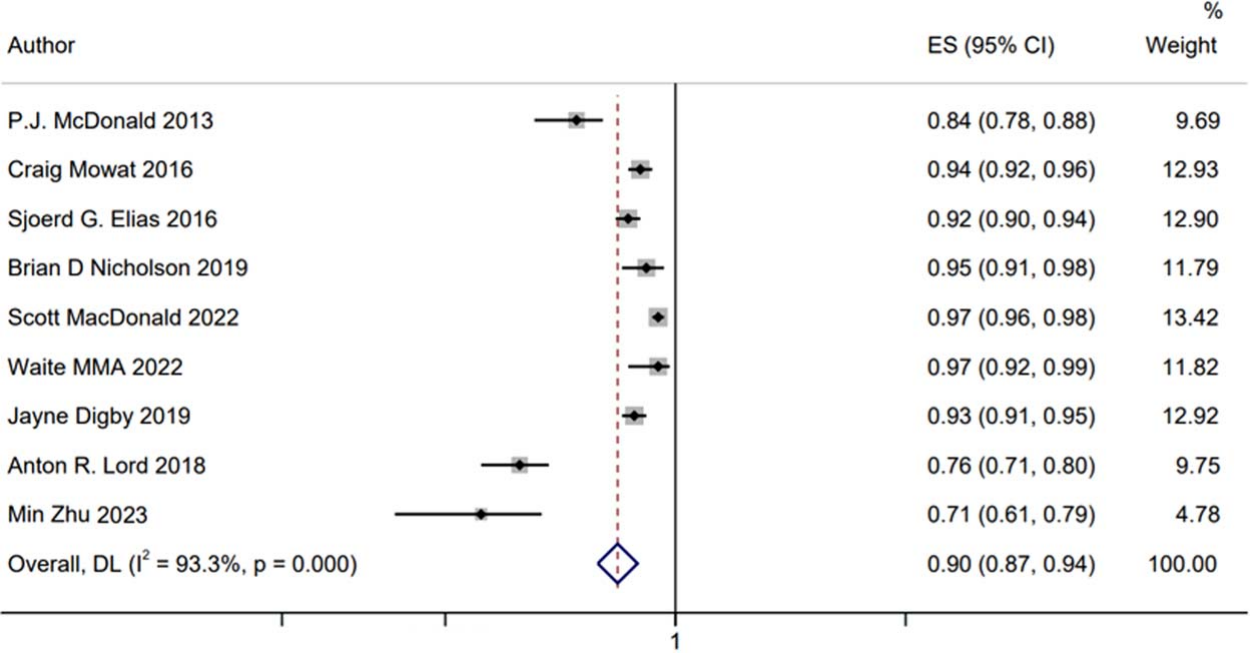
Forest plot of the negative predictive value (NPV) of FIT in SBD screening; the pooled NPV was 0.90 (95% CI: 0.87–0.94), indicating that FIT was a good rule-out test for SBD screening.

**Figure 4:**
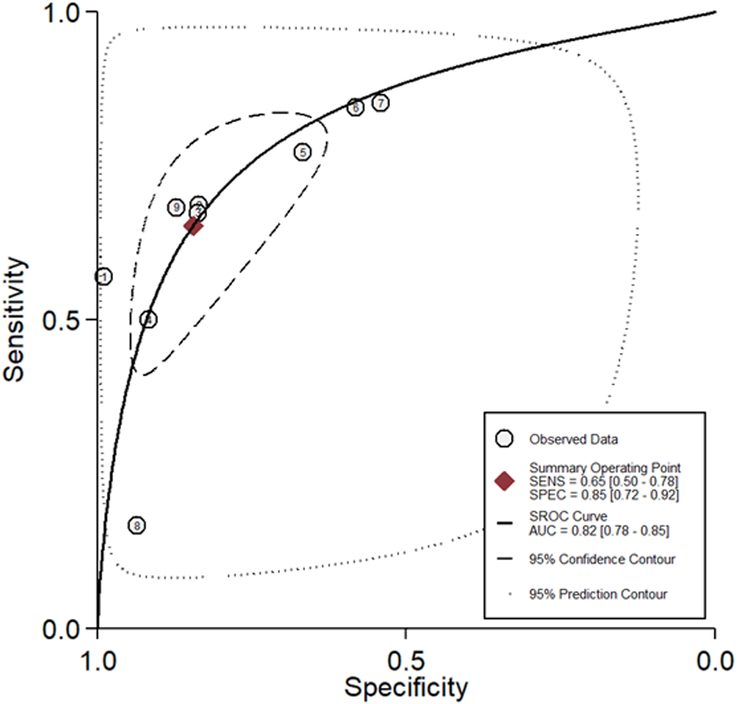
Summary receiver operating characteristic (SROC) plot for evaluation of the accuracy of FIT in SBD screening, and the area under the curve (AUC) was 0.82 (95% CI: 0.78–0.85), implying that FIT has a moderate accuracy in SBD screening.

Our meta-analysis included nine studies with 8615 patients with lower bowel symptoms. The forest plot demonstrated statistically significant heterogeneity in sensitivity, specificity, PLR, NLR, DOR, and NPV. Thus, a random-effects model was used to perform our meta-analysis. After pooling the data from nine trials, the sensitivity, specificity, PLR, NLR, DOR, and NPV of FIT in SBD screening were 0.65 (95% CI: 0.50–0.78), 0.85 (95% CI: 0.72–0.92), 4.2 (95% CI: 2.60–6.90), 0.41 (95% CI: 0.29–0.58), 10 (95% CI: 6–17), and 0.90 (95% CI: 0.87–0.94), respectively. The SROC plot was also conducted to assess the SBD screening accuracy of FIT, the SROC plot showed an area under the curve (AUC) of 0.82 (95% CI: 0.78–0.85); additionally, the Fagan nomogram showed that the posterior probability of SBD was 81% when FIT was positive, while the posterior probability was 29% with a negative FIT, implying that FIT is effective for SBD screening (see details in Figs 2, 3, 4 and Supplemental Fig. 1, Supplemental Digital Content 4, http://links.lww.com/JS9/C893).

### Preplanned subgroup analyses for the ability of FIT in SBD screening stratified by different factors

To explain potential heterogeneity, several preplanned subgroup analyses were conducted under different conditions. For the FIT cut-off values, the pooled sensitivity and specificity of 10 μg Hb/g were set as 0.74 (0.63–0.83) and 0.74 (0.57–0.85), respectively. Lower sensitivity (0.51 [0.29–0.73]) and higher specificity (0.93 [0.83–0.97]) were found for other cut-offs, besides, the cut-off value of 10 μg Hb/g had a higher NPV (0.96 [0.94–0.97]), which means FIT proved to be an excellent rule-out test when adopting 10 μg Hb/g as the threshold. When using CRC, HRA, and IBD in the definition of SBD, the sensitivity and specificity of FIT in SBD screening were 0.64 (0.41–0.82) and 0.79 (0.62–0.89), respectively. In other definitions of SBD, the pooled results showed a summary sensitivity and specificity of 0.65 (0.60–0.70) and 0.87 (0.85–0.89), respectively. The AUC and NPV of FIT for SBD screening were higher for the former definition than for the latter. In the subgroup analyses of FIT combined with other parameters versus FIT alone, a meta-analysis of five studies found the sensitivity and specificity of FIT alone were 0.70 (0.61–0.78) and 0.86 (0.65–0.95), respectively, whereas FIT combined with other parameters showed a sensitivity and specificity of 0.51 (0.29–0.73) and 0.93 (0.83–0.97), respectively. Furthermore, FIT combined with other parameters had a higher AUC but a lower NPV than FIT alone. As for the settings, though the AUC value in primary care and secondary care was similar, FIT in primary care had a higher NPV 0.94 (0.93–0.95) than that in secondary care 0.88 (0.75–0.95) (See details in Table 2).

**Table 2:** Subgroup analysis of the ability of FIT in SBD screening.

Subgroups	Number of articles	Sen	Spe	PLR	NLR	DOR	AUC	NPV
Overall studies	9	0.65 (0.50–0.78)	0.85 (0.72–0.92)	4.2 (2.6–6.9)	0.41 (0.29–0.58)	10 (6–17)	0.82 (0.78–0.85)	0.90 (0.87–0.94)
FIT cut-off								
10 μg Hb/g	5	0.74 (0.63–0.83)	0.74 (0.57–0.85)	2.8 (1.9–4.3)	0.35 (0.27–0.44)	8 (6–11)	0.80 (0.77–0.84)	0.96 (0.94–0.97)
Other cut-offs	4	0.51 (0.29–0.73)	0.93 (0.83–0.97)	7.4 (2.9–19.1)	0.52 (0.33–0.84)	14 (4–48)	0.86 (0.82–0.89)	0.83 (0.72–0.90)
Definition of SBD								
CRC, HRA, IBD	6	0.64 (0.41–0.82)	0.79 (0.62–0.89)	3.1 (2.2–4.3)	0.45 (0.29–0.71)	7 (5–9)	0.79 (0.75–0.82)	0.94 (0.89–0.97)
Other definitions	3	0.65 (0.60–0.70)	0.87 (0.85–0.89)	5.05 (4.21–6.07)	0.40 (0.35–0.47)	21 (7–61)	0.76 (0.68–0.84)	0.88 (0.85–0.90)
FIT combined with other parameters?								
Yes	4	0.51 (0.29–0.73)	0.93 (0.83–0.97)	7.4 (2.9–19.1)	0.52 (0.33–0.84)	14 (4–48)	0.86 (0.82–0.89)	0.86 (0.74–0.93)
FIT alone	5	0.70 (0.61–0.78)	0.86 (0.65–0.95)	5 (2–12.5)	0.35 (0.30–0.41)	14 (6–34)	0.79 (0.75–0.82)	0.95 (0.91–0.97)
Settings								
Secondary care	5	0.62 (0.37–0.82)	0.87 (0.65–0.96)	4.9 (1.9–12.3)	0.44 (0.26–0.74)	11 (4–31)	0.82 (0.78–0.85)	0.88 (0.75–0.95)
Primary care	4	0.68 (0.63–0.74)	0.81 (0.79–0.83)	3.62 (3.20–4.09)	0.39 (0.33–0.46)	11 (8–14)	0.82 (0.78–0.86)	0.94 (0.93–0.95)

AUC, area under the curve; CRC, colorectal cancer; DOR, diagnostic odds ratio; FIT, fecal immunochemical test; HRA, higher-risk adenoma; IBD, inflammatory bowel disease; LRA, lower-risk adenoma; NPV, negative predictive value; NLR, negative likelihood ratio; PLR, positive likelihood ratio; SBD, significant bowel disease; Sen, sensitivity; Spe, specificity.

### Publication bias analysis

A Deeks’ funnel plot was constructed to assess publication bias, and no statistically significant publication bias was observed (*P*=0.33) (Supplemental Fig. 2, Supplemental Digital Content 4, http://links.lww.com/JS9/C893).

## Discussion

This meta-analysis showed that the sensitivity and specificity of FIT in SBD screening were 0.65 (95% CI: 0.50–0.78) and 0.85 (95% CI: 0.72–0.92), respectively, with an AUC of 0.82 (95% CI: 0.78–0.85), and the NPV was up to 0.90 (95% CI: 0.87–0.94). Subgroup analysis demonstrated that FIT was effective in ruling out SBD in the following conditions: with cut-off values 10 μg Hb/g, when the definition of SBD was CRC, HRA, or IBD, when using FIT alone, and in primary care; we believe that FIT could be instrumental in assisting GPs in excluding patients with potential SBD in primary care.

Several methods are currently available for SBD screening. Colonoscopy combined with the pathology of tissue biopsy is regarded as the gold standard for SBD diagnosis; however, it may cause complications such as bleeding and perforations and requires major medical resources^21,22^; colonoscopy is not available at all levels of hospitals, such as the Community Hospital, particularly in developing areas with limited medical resources; therefore, large-scale SBD screening via colonoscopy is unrealistic among all patients with lower bowel symptoms. Fecal biomarker testing and computed tomographic colonography are two important methods of screening for SBD that have been under development for several years. However, they are not yet widely available for various reasons such as CT radiation and uncertainty in screening performance^23^. The fecal occult blood test (FOBT) is a widely used noninvasive screening method currently available for CRC^24^. The FOBT can be divided into two tests based on different principles: the guaiac-based FOBT (gFOBT) and FIT. gFOBT is susceptible to the influence of diet or drugs; thus, the NICE^7^ and British Society of Gastroenterology^25^ guidelines do not recommend gFOBT for CRC screening. Currently, FIT is an accepted method for CRC screening programs worldwide^26^. Most medical laboratories use FIT to perform FOBT. Our meta-analysis only included studies that evaluated the ability of the FIT to screen for SBD. When patients with lower bowel symptoms visit primary care centers, it is important for GPs to assess potential SBD and arrange for further investigation. Previous studies have mainly focused on the value of FIT in the detection of CRC in patients with lower bowel symptoms. Few studies prior to 2019 have addressed SBD as a whole; however, more recently published studies have assessed the ability of FIT in SBD screening in primary care.

Our meta-analysis demonstrated that the FIT is a promising and easy-to-use tool for GPs to rule out SBD in primary care, which has also been verified by several previous primary studies. However, the forest plot in our analysis demonstrated statistically heterogeneity, therefore, a subgroup analysis was conducted, the number of other cut-off values (other than 10 µg Hb/g) reported by the enrolled studies was small, thus, we used the other cut-off values as a whole in the subgroup analysis. Our study revealed that FIT had higher NPV among 10 µg Hb/g compared to other cut-off values. In fact, there is no consensus on the cut-off value of FIT, the cut-off value in the included studies of our meta-analysis ranged from 0 µg Hb/g to 50 µg Hb/g. Normally, a lower FIT cutoff value may cause an increase in sensitivity and a decrease in specificity^27^, and fewer SBDs may be missed; however, more false-positive patients without SBD will be referred for secondary care for endoscopy, aggravating the burden on the medical system. Therefore, an appropriate cutoff value is important. Since the NPV was up to 0.96 (0.94–0.97) in the subgroup analysis of 10 µg Hb/g, we believe 10 µg Hb/g was a good FIT cut-off value to rule out SBD. Our meta-analysis also found that the AUC and NPV of FIT in SBD screening were higher when the definition of SBD only included CRC, HRA, and IBD, previous studies have found that smaller adenomas have a higher possibility of being LRAs^28,29^ and are less likely to induce bleeding, which could also be reflected by the low sensitivity found in the McDonald *et al*.^16^ study that added LRA to the definition of SBD. Several other studies have also shown that the accuracy of FIT is relatively low in detecting the early stages of CRC or adenomas^30–33^. This may explain the lower AUC and NPV in SBD screening when adding LRA to CRC, HRA, and IBD in the definition of SBD; however, only three studies in our subgroup analysis used other definitions of SBD, potentially reducing the reliability of these subgroup results.

It is well documented that fecal hemoglobin concentration increases with age and that males have a higher concentration than females^34–37^, Therefore, it is inevitable that FIT would be affected by these unchangeable interference factors. Currently, risk prediction models are widely applied to improve diagnostic accuracy, and a prediction model that includes both FIT and other variables may potentially decrease the effect of these unchangeable interference factors. Lord *et al*.^19^. combined FIT with clinical and demographic data to develop a risk prediction model called the risk assessment tool (RAT) and demonstrated that RAT had a better diagnostic accuracy for SBD screening than FIT alone; in 2016, Elias *et al*.^18^ and Zhu *et al*.^20^ drew a similar conclusion. However, Digby *et al*.^17^ found that the FAST score did not enhance the utility of f-Hb alone. Our subgroup analysis enrolled these four studies and found a higher AUC of FIT combined with other variables in SBD screening when compared to that in FIT alone, our study also revealed the NPV was up to 0.95 (0.91–0.97) for FIT alone while the NPV lower to 0.86 (0.74–0.93) when FIT combined with other variables. A possible explanation may help interpret this result: the four enrolled studies used different combined variables and FIT cutoff values, which may have caused heterogeneity and further influenced the accuracy of the subgroup analysis results of FIT combined with other variables; however, it is noteworthy that FIT alone may be sufficient to rule out SBD for high NPV. The application of artificial intelligence (AI) in the medical field is rapidly developing, and machine learning (ML) is a major area^38^. In clinical practice, ML prediction models may be more accurate than traditional risk prediction models for disease^39^. Currently, no ML prediction model has been published for detecting or ruling out SBD as a whole; thus, we believe that an ML prediction model combining FIT with other variables may be promising for achieving higher accuracy for SBD screening.

Our meta-analysis has several advantages. To the best of our knowledge, this is the first meta-analysis to assess the performance of the FIT in SBD screening among patients with lower bowel symptoms. Secondly, the results of our instructive subgroup analysis may be applicable to primary care settings. Third, although only nine studies were included, the number of patients was 8615, strengthening the reliability of the results. Furthermore, our meta-analysis strictly adhered to the broad EQUATOR guidelines to guarantee scientific reliability^40^. However, certain limitations of this study must be addressed. First, most of the included studies were conducted in Europe and Australia; thus, the results may not be applicable to populations outside. Second, heterogeneity existed among the included studies; although we conducted a subgroup analysis to identify sources of heterogeneity, other factors, such as the time to conduct FIT and follow-up time of patients with lower bowel symptoms, may also cause heterogeneity. Third, although FIT is useful in ruling out SBD, no SBD screening method is perfect; a minority of SBD cases will remain undetected when using FIT alone, and the current practice does not recommend basing decisions solely on FIT results. GPs should be strongly encouraged, especially in patients with persistent symptoms, to not rely solely on FIT or even decide on a second-level analysis, considering that FIT may lead to delayed diagnoses, particularly in cases of false negatives^41^. Finally, although this study found that FIT had great application value in ruling out SBD, the use of antiplatelet and anticoagulant drugs^42^ and other benign lesions, such as hemorrhoids^43^ may also result in positive FIT results, leading to an increase in the false positive rate (FPR) of FIT in SBD screening. Since FIT combined with other variables showed a higher PLR than did FIT alone in the subgroup analysis, we thought that the development of an SBD screening model or score that considered both FIT and other key variables might be a good idea to decrease FPR in SBD screening in the future. Importantly, due to the lack of doctors in some countries^44–46^, the increasingly early onset of colorectal tumors^47,48^, the greater frequency of neuroendocrine tumors^49^ and chronic intestinal inflammatory diseases^50,51^, screening for SBD might be more challenging, thus, it is worth emphasizing that patients need to be referred to gastroenterologists for further diagnosis and treatment when necessary, in particular when the GPs find it difficult to assess the patient’s condition in daily clinical practice^52–55^. Nevertheless, these limitations do not alter the fact that the FIT is worthy of attention in SBD screening among patients with lower bowel symptoms, especially for those with inexperienced GPs, which provides an objective and easy-to-use way to rule out SBD.

## Conclusion

In conclusion, this study demonstrated that FIT is likely to be a good rule-out test for GPs for SBD screening in patients with lower bowel symptoms. Of note, no SBD screening method is perfect, minority of SBD cases will remain undetected when using FIT alone, to avoid diagnostic delay, it is also quite important for GPs to promptly refer patients to gastroenterologists when necessary. In the future, additional high-quality studies that consider both FIT and other key variables are needed to further improve the accuracy of SBD screening.

## Ethical approval

There is no need for approval of ethics committee or institutional review board, because this study is a meta-analysis which do not involve any clinical trials and patient consent.

## Consent

Informed consent was waived since the data of this study were all from public database and the patients’ personal information was anonymized.

## Source of funding

The National Natural Science Foundation of China (No. 82170549) funded this manuscript.

## Author contribution

J.Z. and F.L.: conception and design; W.D.: administrative support; F.L. and J.Z.: provision of study materials or patients; J.Z. and H.X.: collection and assembly of data; C.T. and F.L.: data analysis and interpretation. All authors contributed in manuscript writing and final approval of manuscript.

## Conflicts of interest disclosure

The authors have no conflicts of interest to declare.

## Research registration unique identifying number (UIN)

CRD42022353328.

## Guarantor

Weiguo Dong.

## Data availability statement

Data sharing is not applicable to this article, as this study is a meta-analysis.

## Provenance and peer review

Not commissioned, externally peer-reviewed.

## Supplementary Material
