# Supplementary material for: Negative predictive value of fecal immunochemical testing in significant bowel disease screening: a systematic review and meta-analysis
Source: Int J Surg. 2024 Jun 26;111(1):1182–90. doi: 10.1097/JS9.0000000000001844 (PMC11745644; doi:10.1097/JS9.0000000000001844)
Supplement: Supplementary file 2 [file js9-111-1182-s002.docx]

**Identification of studies via databases and registers**

Records identified from Pubmed, Embase, and Cochrane:

Databases (n =7438)

Records removed *before screening*:

Duplicate records removed

(n = 2672)

**Identification**

Records excluded

with reasons:
1344 Reviews
1569 Coverage not matched
1280 Only investigated a specific gut disease
463 Editorials or letters
79 Paediatric patients

(n = 4735)

Records screened

(n =4766)

Reports sought for retrieval

(n = 31)

Reports not retrieved

(n =0)

**Screening**

Reports assessed for eligibility

(n =31)

Reports excluded:

Lacking in data required for analysis or just a report of study protocol (n =22)

Studies included in review

(n = 9)

Reports of included studies

(n = 9)

**Included**

*Consider, if feasible to do so, reporting the number of records identified from each database or register searched (rather than the total number across all databases/registers).

**If automation tools were used, indicate how many records were excluded by a human and how many were excluded by automation tools.

*From:*  Page MJ, McKenzie JE, Bossuyt PM, Boutron I, Hoffmann TC, Mulrow CD, et al. The PRISMA 2020 statement: an updated guideline for reporting systematic reviews. BMJ 2021;372:n71. doi: 10.1136/bmj.n71

For more information, visit: http://www.prisma-statement.org/
