# Supplementary material for: Negative predictive value of fecal immunochemical testing in significant bowel disease screening: a systematic review and meta-analysis
Source: Int J Surg. 2024 Jun 26;111(1):1182–90. doi: 10.1097/JS9.0000000000001844 (PMC11745644; doi:10.1097/JS9.0000000000001844)
Supplement: Supplementary file 4 [file js9-111-1182-s004.docx]

**Supplementary Material:**

**Supplementary Table 1**. The detailed characteristics of the included studies.

**Supplementary Table 2**. Methodological quality assessment of included studies. Six were judged as having a low risk of bias in the item of patient selection^1-4,7,9^, two studies^6,8^ were unclear risk and one study^5^ was high risk. All enrolled studies were at a low risk of bias in the index test, reference standard and flow and timing. As for the items in applicability concerns, all studies were judged as low concern.

**Supplemental Figure 1**. The fagan nomogram showed the posterior probability of SBD was 81% when the FIT was positive, while the posterior probability was 29% with a negative FIT, demonstrating that FIT has the ability to screen for SBD.

**Supplemental Figure 2**. The Deeks’ funnel plot to evaluate the potential publication bias, this funnel plot demonstrated no statistically significant publication bias in this meta‐analysis (*P*=0.33).

**Literature Search Strategies:**

((((significant colorectal disease[Title/Abstract]) OR (significant bowel disease[Title/Abstract])) OR ((((((((((((colorectal cancer[Title/Abstract]) OR (colorectal neoplasm[Title/Abstract])) OR (colorectal carcinoma[Title/Abstract])) OR (colorectal tumor[Title/Abstract])) OR (inflammatory bowel disease[Title/Abstract])) OR (Crohn[Title/Abstract])) OR (Crohns-disease[Title/Abstract])) OR (ulcerative colitis[Title/Abstract])) OR (IBD[Title/Abstract])) OR (CD[Title/Abstract])) OR (UC[Title/Abstract])) OR (adenoma[Title/Abstract]))) AND (((((((((faecal immunochemical test[Title/Abstract]) OR (immunochemical faecal occult blood test[Title/Abstract])) OR (FIT[Title/Abstract])) OR (occult blood[Title/Abstract])) OR (fecal occult blood test[Title/Abstract])) OR (faecal occult blood test[Title/Abstract])) OR (fob[Title/Abstract])) OR (fobt[Title/Abstract])) OR (ifobt[Title/Abstract]))) AND ((((screening[Title/Abstract]) OR (screen[Title/Abstract])) OR (detect[Title/Abstract])) OR (detection[Title/Abstract]))

**Supplemental Table 1: The detailed characteristics of included studies**

| **Study** | **FIT cut-off** | **FIT combined with other parameters?** | **TP** | **FP** | **TN** | **FN** | **Sen** | **Spe** | **NPV** |
| --- | --- | --- | --- | --- | --- | --- | --- | --- | --- |
| P.J. McDonald 2013 | 51 ng Hb/ml | FIT alone | 49 | 2 | 192 | 37 | 0.57 | 0.99 | 0.84 |
| Craig Mowat 2016 | 10 ug Hb/g | FIT alone | 70 | 107 | 546 | 32 | 0.68 | 0.84 | 0.95 |
|  | 0 ug Hb/g | FIT alone | 90 | 350 | 303 | 12 | 0.88 | 0.46 | 0.96 |
| Sjoerd G. Elias 2016 | 6 ug Hb/g | FIT alone | 95 | 109 | 560 | 46 | 0.67 | 0.84 | 0.92 |
|  | 6 ug Hb/g | FIT+ routine clinical data | 131 | 429 | 240 | 10 | 0.93 | 0.36 | 0.96 |
| Brian D Nicholson 2019 | 7 ug Hb/g | FIT alone | 11 | 20 | 198 | 9 | 0.55 | 0.91 | 0.96 |
|  | 10 ug Hb/g | FIT alone | 10 | 18 | 200 | 10 | 0.50 | 0.92 | 0.95 |
|  | 20 ug Hb/g | FIT alone | 9 | 13 | 205 | 11 | 0.45 | 0.94 | 0.95 |
|  | 50 ug Hb/g | FIT alone | 8 | 6 | 212 | 12 | 0.40 | 0.97 | 0.95 |
| Scott MacDonald 2022 | 10 ug Hb/g | FIT alone | 253 | 1269 | 2540 | 75 | 0.77 | 0.67 | 0.97 |
| Waite MMA 2022 | 10 ug Hb/g | FIT alone | 27 | 105 | 146 | 5 | 0.84 | 0.58 | 0.97 |
| Jayne Digby 2019 | 10 ug Hb/g | FIT alone | 252 | 528 | 623 | 44 | 0.85 | 0.54 | 0.93 |
|  | 10 ug Hb /g | f-Hb + age + sex test score (FAST score) | 286 | 857 | 294 | 10 | 0.97 | 0.26 | 0.97 |
| Anton R. Lord 2018 | 0 ug Hb/g | FIT alone | 19 | 20 | 294 | 95 | 0.17 | 0.94 | 0.76 |
|  | 0 ug Hb/g | FIT+ routine clinical data | 28 | 11 | 303 | 86 | 0.25 | 0.96 | 0.78 |
| Min Zhu 2023 | 22.5 ng Hb /mL | FIT alone | 73 | 12 | 82 | 34 | 0.68 | 0.87 | 0.71 |
|  | 50 ng Hb /mL | FIT alone | 64 | 5 | 89 | 43 | 0.60 | 0.95 | 0.67 |
|  | 50 ng Hb /mL | FIT+ FC | 87 | 26 | 68 | 20 | 0.81 | 0.72 | 0.77 |

FIT: Faecal immunochemical test; TP: True positive; FP: False positive; TN: True negative; FN: False negative; Sen: Sensitivity; Spe: Specificity; NPV: Negative predictive value; FC: fecal calprotectin.

**Supplemental Table 2: Methodological quality assessment of included studies**

| **Study** | **Risk of bias** | | | | **Applicability concerns** | | |
| --- | --- | --- | --- | --- | --- | --- | --- |
|  | **Patient selection** | **Index test** | **Reference standard** | **Flow and timing** | **Patient selection** | **Index test** | **Reference standard** |
| P.J. McDonald 2013 | LR | LR | LR | LR | LC | LC | LC |
| Craig Mowat 2016 | LR | LR | LR | LR | LC | LC | LC |
| Sjoerd G. Elias 2016 | LR | LR | LR | LR | LC | LC | LC |
| Brian D Nicholson 2019 | LR | LR | LR | LR | LC | LC | LC |
| Scott MacDonald 2022 | LR | LR | LR | LR | LC | LC | LC |
| Waite MMA 2022 | HR | LR | LR | LR | LC | LC | LC |
| Jayne Digby 2019 | UR | LR | LR | LR | LC | LC | LC |
| Anton R. Lord 2018 | UR | LR | LR | LR | LC | LC | LC |
| Min Zhu 2023 | LR | LR | LR | LR | LC | LC | LC |

LR: low risk; HR: high risk; UR: unclear risk; LC: low concern; HC: high concern; UC: unclear concern.

**
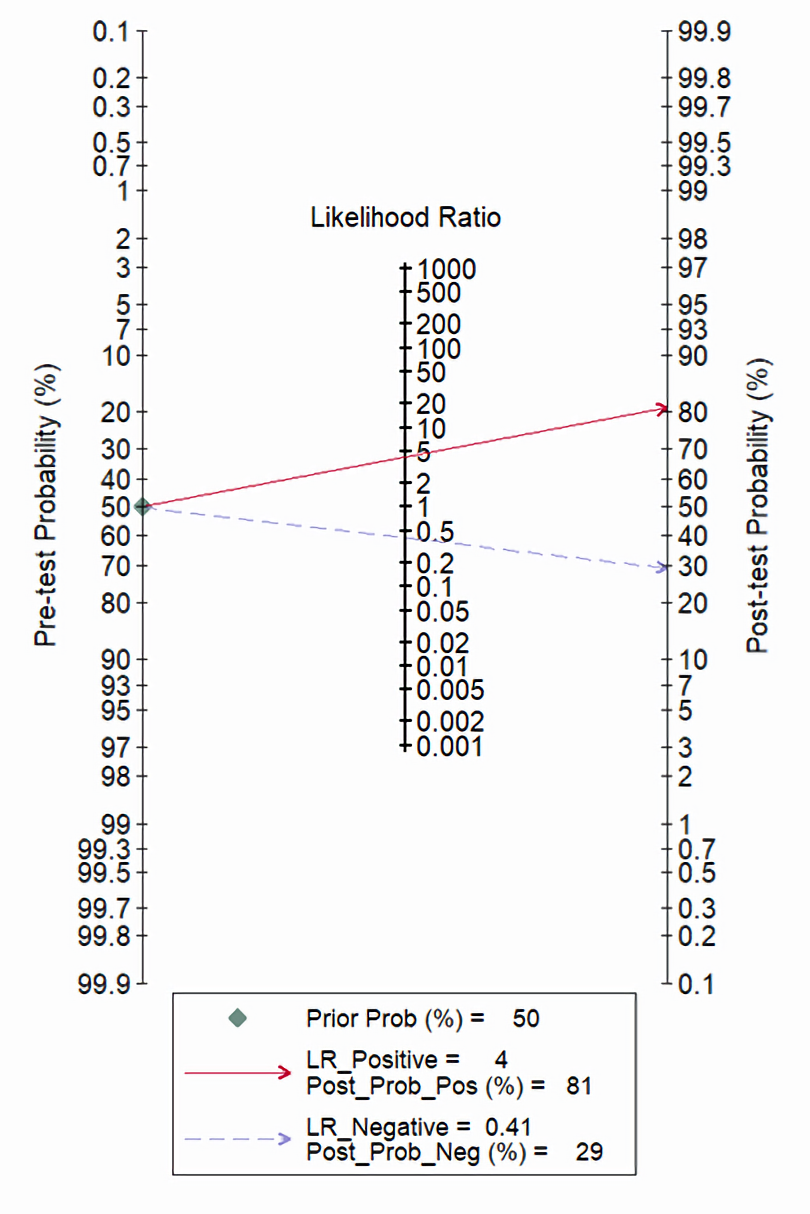
**

**Supplemental Figure 1**. The fagan nomogram.

**
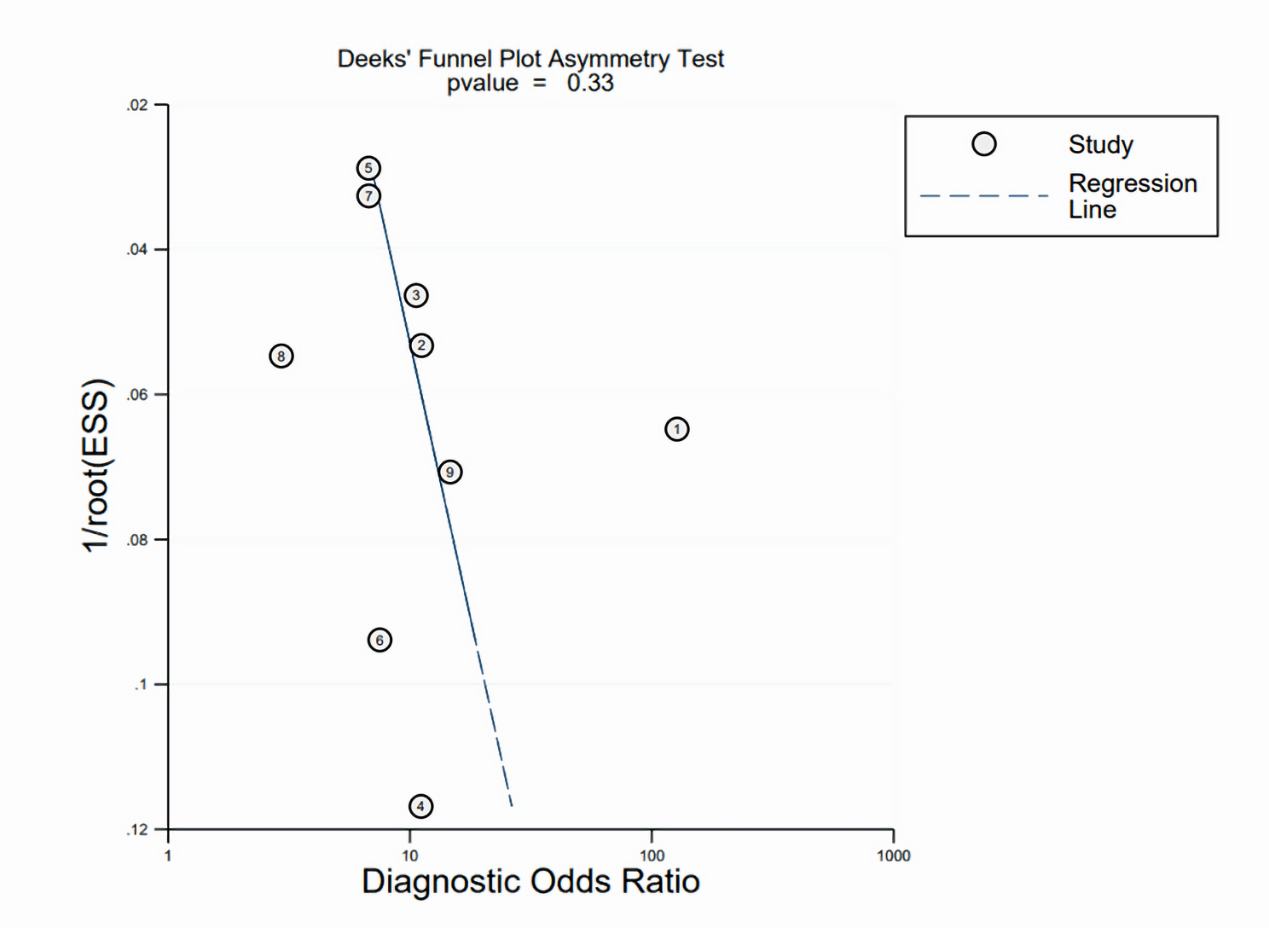
**

**Supplemental Figure 2**. The Deeks’ funnel plot to evaluate the potential publication bias
